# Prognostic values of the Berlin definition criteria, blood lactate level, and fibroproliferative changes on high-resolution computed tomography in ARDS patients

**DOI:** 10.1186/s12890-019-0803-0

**Published:** 2019-02-11

**Authors:** Tetsuro Kamo, Sadatomo Tasaka, Takeshi Suzuki, Takanori Asakura, Shoji Suzuki, Kazuma Yagi, Ho Namkoong, Makoto Ishii, Hiroshi Morisaki, Tomoko Betsuyaku

**Affiliations:** 1 Division of Pulmonary Medicine, Department of Medicine, Keio University School of Medicine, Tokyo, 160-8582 Japan; 2 Department of Respiratory Medicine, Hirosaki University Graduate School of Medicine, 5 Zaifucho, Hirosaki, 036-8562 Japan; 3 Department of Anesthesiology, Keio University School of Medicine, Tokyo, 160-8582 Japan

**Keywords:** ARDS, Berlin definition, High-resolution computed tomography, Lactate, Mortality

## Abstract

**Background:**

In the Berlin definition, acute respiratory distress syndrome (ARDS) is stratified into three stages according to oxygenation severity at the onset. The relevance between ARDS severity and prognosis varies among published reports and has not been verified, especially in Asian patients.

**Methods:**

In this study, we examined the associations between the Berlin definition criteria and prognosis and clinical parameters, including high-resolution computed tomography (HRCT) scores of fibroproliferative changes of the lungs. One hundred fifty-three patients (45 females; mean age, 67 y/o), who met the Berlin definition and received treatment in our intensive care unit between January 2012 and December 2015, were enrolled.

**Results:**

The severity of ARDS was mild in 42 patients, moderate in 71, and severe in 40. The underlying diseases included pneumonia in 56 patients and aspiration in 43. Forty-two (27.5%) patients were deceased within 30 days, and the 30-day mortality was 10% in mild ARDS, 23% in moderate, and 55% in severe, which were significantly different (*P* <  0.05). In the non-survivors, APACHE II, SOFA, and SAPS II scores were higher than in the survivors (*P* <  0.001). Multivariate analyses revealed that elevated blood lactate level (≥ 2.0 mmol/L) and increased HRCT scores were significantly associated with weaning failure and 30-day mortality of the patients with ARDS.

**Conclusions:**

These results suggested that the severity criteria in the Berlin definition might be associated with the prognosis of the patients. Blood lactate levels and HRCT score might be predictive of the outcome of patients with ARDS.

## Introduction

Acute respiratory distress syndrome (ARDS) is an acute inflammatory lung injury, characterized by increases in pulmonary vascular permeability and extravascular lung water and loss of aerated lung areas [1]. The Berlin definition of the 2012 announcement defines the severity of ARDS in the oxygenation index [1]. The new PaO_2_/FIO_2_ thresholds chosen for different levels of ARDS severity were reported to be helpful in categorizing patients with respect to different approaches and mortality increased with stages of ARDS from mild to moderate to severe [1, 2]. In another report, however, the severity of respiratory failure was not significantly associated with patient death at day 28 [3].

In the recent Large Observational Study to Understand the Global Impact of Severe Acute Respiratory Failure (LUNG SAFE) study, which included 29,144 patients from 459 intensive care units (ICUs) in 50 countries, there was a decreased likelihood of survival at day 28 with increasing severity [4]. The relevance between ARDS severity and prognosis is variable among published reports and has not been verified enough, especially in Asian patients. Recently, Maiolo and colleagues reported that using the 150-mm-Hg PaO_2_/FIO_2_ threshold gave a more homogeneous distribution of patients with ARDS across the severity subgroups and identified two populations that differed in their anatomical and physiological characteristics [5]. In addition, lung recruitability and [^18^F]2-fluoro-2-deoxy-D-glucose uptake quantified with a positron emission tomography were associated with ARDS severity, indicating that lung imaging could be helpful to estimate the disease severity [6].

Computed tomography and other lung imaging have significantly changed our understanding and management of ARDS [7]. Compared to conventional CT scan, high-resolution CT (HRCT) is superior in demonstrating pathological changes of the lungs. HRCT findings are known to represent the pathologic phases of diffuse alveolar damage [8, 9]. For example, traction bronchiectasis, which is associated with loss of lung volume, suggests pulmonary fibroproliferation, which is predictive of increased mortality with an increased susceptibility to multiple organ failure, along with ventilator dependency and its associated outcomes [10]. However, it remains to be examined whether the HRCT scores of fibroproliferative changes are associated with the ARDS severity by the Berlin definition.

In the present study, we evaluated whether the severity criteria by the Berlin definition was associated with the outcome in consecutive patients with ARDS in an ICU of a university hospital. We also assessed prognostic values of other clinical parameters including serum markers and HRCT scores of fibroproliferative changes of the injured lungs.

## Methods

### Study design

This is a retrospective observational study conducted in a general ICU of our Keio University hospital. We evaluated the medical records of 2071 consecutive patients who stayed in the ICU between January 2012 and December 2015. Patients who were resuscitated from cardiopulmonary arrest before the ICU admission were excluded. The study protocol was approved by the ethics committee of Keio University School of Medicine (approval number: 20160010).

### Identification of ARDS

The diagnosis of ARDS was made when a patient met the Berlin definition criteria: (1) presence of acute hypoxemic respiratory failure, (2) onset within 7 days of insult, or new (within 7 days) or worsening respiratory symptoms; (3) bilateral opacities on chest x-ray or CT not fully explained by effusions, lobar or lung collapse, or nodules; and (4) cardiac failure not the primary cause of acute respiratory failure.

### Data collection

The following data were recorded at ICU admission for the ARDS patients: age, sex, body mass index (BMI), and Simplified Acute Physiology Score (SAPS) II score. We put the medical history, lung images, and laboratory tests together and judged the underlying diseases. The following data were collected at the time of inclusion: underlying disease, time to onset, settings of invasive or noninvasive mechanical ventilation, arterial blood gases including blood lactate level, complete blood counts, and serum levels of bilirubin, creatinine, C-reactive protein (CRP) and lactate dehydrogenase (LDH). In addition, the worst values of Sequential Organ Failure Assessment (SOFA) score and Acute Physiology and Chronic Health Evaluation (APACHE) II score during the first 24 h after the admission were also recorded.

### HRCT examination and scoring

The patients underwent whole lung HRCT scanning within 48 h of ICU admission using a multidetector-row CT scan (Aquilion ONE™, Toshiba Medical Systems, Tochigi, Japan). All HRCT images were obtained with 2-mm thickness and 15-mm table speed per rotation and were performed at full inspiration from the lung apex to base. We carefully excluded patients with pre-existing chronic interstitial lung diseases from the HRCT analysis by history taking, imaging data available before onset of ARDS and the presence of coarse reticulation and honeycombing on HRCT scans suggesting of chronic pulmonary fibrosis. In case honeycombing was detected in previous HRCT, the patient was excluded from HRCT analysis. The HRCT images from 140 patients were subjected to analysis. HRCT findings were graded on a scale of 1–6 based on the classification system correlating with previously described pathology [9]: 1, normal attenuation; 2, ground-glass attenuation; 3, consolidation; 4, ground-glass attenuation with traction bronchiolectasis or bronchiectasis; 5, consolidation with traction bronchiolectasis or bronchiectasis and 6, honeycombing. The presence of each of these six abnormalities was assessed independently in three (upper, middle and lower) zones of each lung. The upper zone was defined as the area above the level of the carina, the middle zone as the area between the level of the carina and that of the infrapulmonary vein and the lower zone as the area below the level of the infrapulmonary vein. The extent of each abnormality was determined by visually estimating the percentage (to the nearest 10%) of the affected lung parenchyma in each zone. The assessments of the two observers were averaged. The abnormality score for each zone was calculated by multiplying the percentage area by the point value (1–6). The six zone scores were averaged to determine the total score for each abnormality in each patient. The overall HRCT score for each patient was obtained by adding the six averaged scores.

### Statistical analysis

Data are expressed as the mean value with the standard deviation (SD). For multiple comparisons, analysis of variance was performed with a Bonferroni correction to determine significant differences. Univariate and multivariate logistic regression analyses were performed in order to assess the effects of factors on ventilator weaning failure and 30-day mortality. All statistical analyses were performed with EZR (Saitama Medical Center, Jichi Medical University, Saitama, Japan), which is a graphical user interface for R (The R Foundation for Statistical Computing, Vienna, Austria). The difference was considered to be statistically significant if the *P* value is less than 0.05.

## Results

### Patient demographics

Among 2071 patients evaluated, 153 (7.4%) met the Berlin definition criteria of ARDS. Forty-five (29%) patients were female and mean age was 67 years old. The most frequent underlying disease was pneumonia (37%), followed by aspiration (28%) and sepsis (6.5%). Concomitant diseases included diabetes (23%), chronic respiratory disease (21%), and chronic kidney disease (20%).

### Severity of ARDS

The severity of ARDS was stratified in accordance with the Berlin definition criteria. ARDS was mild in 42 (27.5%) patients, moderate in 71 (46.4%), and severe in 40 (26.1%). We compared patient demographics, comorbidities, and clinical parameters, such as vital signs, use of catecholamine, APACHE II and SOFA scores, laboratory data, and the HRCT scores between patients with mild, moderate, and severe ARDS (Table 1). Although patient demographics and comorbidities did not differ, there were significant differences in APACHE II and SOFA scores, blood lactate levels, and HRCT scores between the groups (*P* <  0.01). In addition, ventilatory variables on the first days of ARDS were also significantly different between the groups (*P* <  0.05).

**Table 1:** Demographics, comorbidities, and clinical parameters of the study subjects

	Mild (*n* = 42)	Moderate (*n* = 71)	Severe (*n* = 40)	*P* value
Male gender, n (%)	28 (66.7)	49 (69.0)	31 (77.5)	0.49
Age, mean ± SD (y)	66.7 ± 16.8	68.1 ± 16.2	65.3 ± 16.9	0.7
BMI, mean ± SD (kg/m^2^)	20.4 ± 4.0	20.5 ± 4.5	20.6 ± 4.2	0.97
Body temperature	37.4 ± 0.9	37.7 ± 1.4	37.5 ± 2.6	0.64
Underlying pneumonia (bacterial/aspiration/VAP^a^)	8/17/4	28/19/1	20/7/0	
Comorbidity				
chronic heart disease	25%	24%	23%	0.94
chronic respiratory disease	13%	18%	34%	0.30
chronic kidney disease	19%	18%	25%	0.71
glucose intolerance	24%	21%	25%	0.88
connective tissue disease	2%	10%	15%	0.14
Direct lung injury	71%	87%	78%	0.095
Sepsis	81%	62%	58%	0.045
Use of catecholamine	36%	39%	58%	0.91
Immunosuppressant usage	22%	16%	18%	0.71
Clinical scores				
APACHE II score	20.9 ± 6.5	23.6 ± 5.8	27.7 ± 8.4	< 0.001
SOFA score	9.3 ± 3.1	10.7 ± 3.0	12.8 ± 3.9	< 0.001
SAPS II score	55.4 ± 13.9	61.5 ± 13.5	65.9 ± 16.8	0.059
Laboratory data				
White blood cell (× 10^3^/mm^3^)	16.1 ± 8.3	13.1 ± 8.1	13.4 ± 12.6	0.97
Platelet (× 10^3^/mm^3^)	209 ± 135	196 ± 146	171 ± 152	0.25
Total bililubin (mg/dL)	2.1 ± 4.4	1.6 ± 3.6	2.8 ± 7.0	0.18
Creatinine (mg/dL)	1.4 ± 1.4	1.4 ± 1.3	1.9 ± 1.6	0.15
C-reactive protein (mg/dL)	10.6 ± 9.0	12.2 ± 10.4	14.3 ± 12.1	0.48
LDH (IU/L)	334 ± 181	339 ± 182	436 ± 259	0.078
HCO_3_^−^ (mEq/L)	24.9 ± 10.4	25.9 ± 6.7	25.5 ± 9.1	0.83
PaCO_2_ (Torr)	41.0 ± 9.6	47.4 ± 18.8	46.7 ± 15.7	0.1
Lactate (mmol/L)	1.7 ± 1.2	2.5 ± 2.8	3.7 ± 4.2	0.004
HRCT score	151 ± 43	182 ± 71	204 ± 80	0.005
Ventilatory variables				
Max. inspiratory pressure (cmH_2_O)	17.0 ± 7.7	20.5 ± 6.8	21.4 ± 6.9	0.012
Respiratory rate (breaths/min)	23 ± 6	27 ± 8	28 ± 10	0.007
PEEP (cmH_2_O)	8 ± 3	10 ± 4	11 ± 4	0.002
FiO_2_	0.43 ± 0.10	0.46 ± 0.10	0.5 ± 0.12	0.023
Outcomes				
Ventilator-free days	18.0 ± 8.1	11.5 ± 9.6	6.2 ± 8.8	< 0.001
ICU free days	14.7 ± 8.2	11.9 ± 8.4	5.8 ± 7.5	< 0.001
30-day mortality (%)	4 (10%)	16 (23%)	22 (55%)	0.012

^a^ventilator-associated pneumonia

### Patient prognosis

**Fig. 1:**
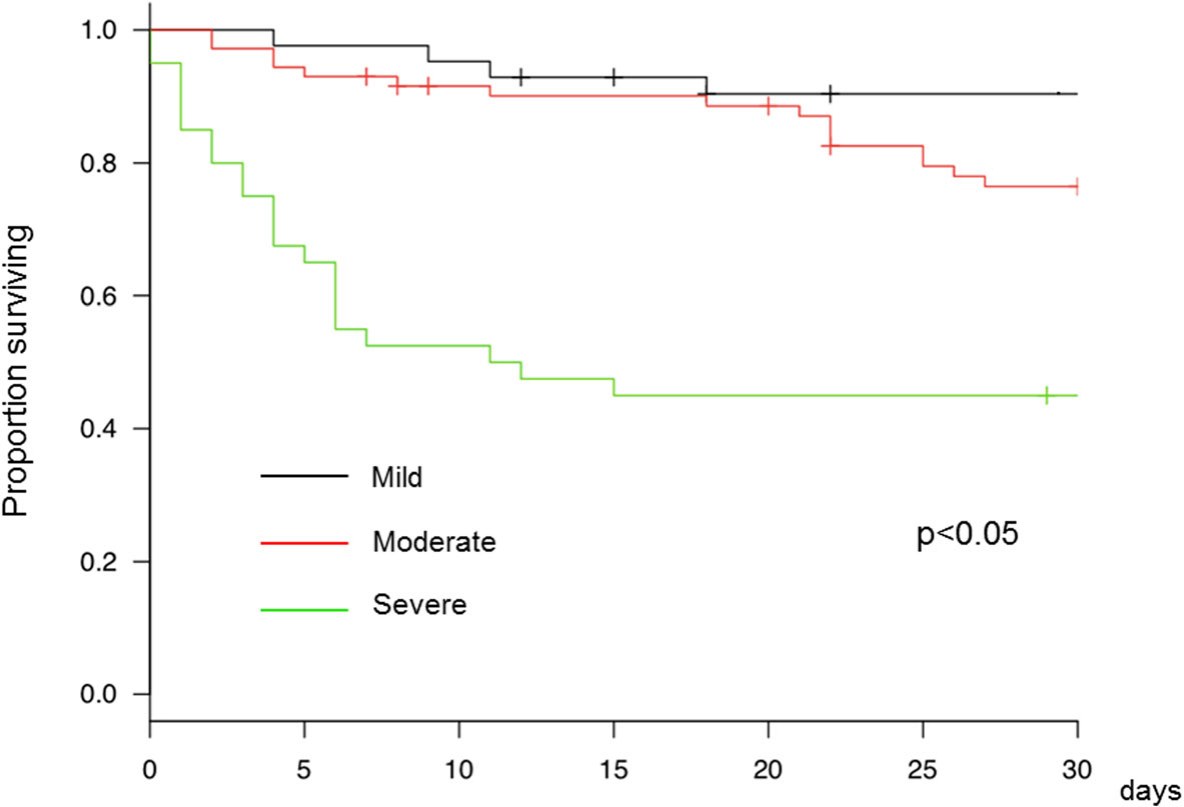
Kaplan-Meier curves for the patient survival

Among 153 patients with ARDS, 42 (27.5%) and 50 (32.7%) were deceased within 30 and 60 days, respectively. The 30-day mortalities of patients with mild, moderate, and severe ARDS were 9.5, 22.5, 55.0%, respectively. There were significant differences in the 30-day mortalities between the groups (*P* = 0.012). The Kaplan-Meier curves for the patient survival after ICU admission was shown in Fig. 1. Whereas the survival of the patients with severe ARDS was apparently worse, the survival curves of those with mild and moderate ARDS were close. The severity stratification in the Berlin definition may be useful for identifying severe ARDS patients with high risk of death, but it may have less significance to differentiate between mild and moderate diseases.

We compared patient demographics, comorbidities, and clinical parameters, such as vital signs, severity of ARDS, use of catecholamine, APACHE II, SOFA, and SPAS II scores, serum markers, and the HRCT scores between patients who survived on day 30 and those who did not (Table 2). The non-survivors were characterized by older age, higher body temperature, and more impaired oxygenation. In the non-surviving patients, APACHE II, SOFA, and SAPS II scores were also higher than in the survivors (*P* < 0.001). In addition, the non-survivors showed decreased platelet counts and elevated levels of serum creatinine and blood lactate compared with the survivors, suggesting multiple organ failure and tissue hypoxia. The HRCT scores were not significantly different between them.

**Table 2:** Demographics, comorbidities, and clinical parameters of survivors and non-survivors

Parameters	Surviving (*n* = 113)	Non-surviving (*n* = 40)	*P* value
Male gender, n (%)	74 (65.5)	34 (85%)	0.0256
Age, mean ± SD (y)	61.7 ± 15.1	68.9 ± 16.6	0.0162
BMI, mean ± SD (kg/m^2^)	20.2 ± 4.3	21.6 ± 4.1	0.0831
Body temperature	37.3 ± 1.7	38.4 ± 1.3	0.00465
Underlying pneumonia (bacterial/aspiration /VAP^a^)	42/34/3	15/9/1	0.922
Comorbidity			
chronic heart disease	26%	18%	0.29
chronic respiratory disease	21%	20%	0.98
chronic kidney disease	20%	20%	1.00
glucose intolerance	22%	25%	0.83
connective tissue disease	8%	13%	0.52
Direct lung injury	81%	80%	1.00
Berlin definition severity			
mild	38	4	
moderate	56	15	< 0.001
severe	19	21	
Sepsis	70%	55%	0.12
Use of catecholamine	42%	25%	0.086
Clinical scores			
APACHE II score	23 ± 7	28 ± 7	< 0.001
SOFA score	10 ± 3	13 ± 4	< 0.001
SAPS II score	58 ± 14	68 ± 14	< 0.001
Laboratory data			
White blood cell (×10^3^/mm^3^)	14.0 ± 7.9	13.9 ± 13.3	0.95
Platelet (× 10^3^/mm^3^)	208 ± 154	152 ± 106	0.035
Total bililubin (mg/dL)	2.0 ± 4.9	2.1 ± 4.8	0.957
Creatinine (mg/dL)	1.4 ± 1.2	2.0 ± 1.9	0.035
C-reactive protein (mg/dL)	12.1 ± 9.8	12.9 ± 12.3	0.659
LDH (IU/L)	377 ± 228	324 ± 133	0.163
HCO3- (mEq/L)	25.9 ± 7.9	24.3 ± 9.8	0.29
PaCO2 (Torr)	44.5 ± 15.8	48.2 ± 16.9	0.216
Lactate (mmol/L)	2.4 ± 2.8	3.7 ± 3.1	< 0.001
HRCT score	175 ± 69	193 ± 71	0.182
Max inspiratory pressure (cmH_2_O)	18 ± 7	23 ± 6	< 0.001

^a^ventilator-associated pneumonia

### Prognostic value of the HRCT score

**Fig. 2:**
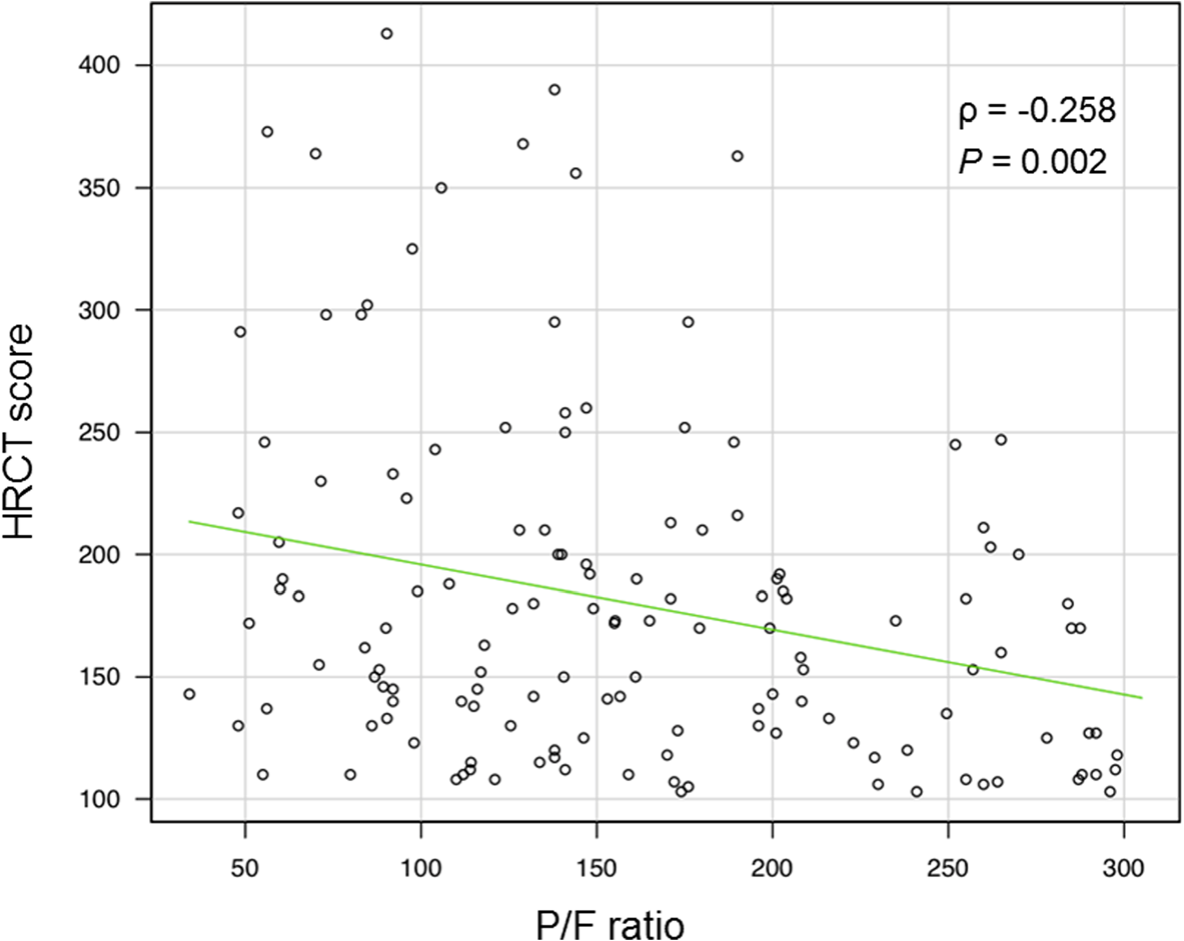
Correlation between the HRCT score and PaO_2_/FIO_2_ ratio. The HRTCT score was inversely correlated with PaO_2_/FIO_2_ ratio (ρ = − 0.258, *P* = 0.002)

The concordance rate between the two observers was 91%. In case of major discrepancy, the score was corrected after discussion. The overall HRCT scores were 151 ± 43 in mild ARDS patients, 182 ± 71 in moderate, and 204 ± 80 in severe (Table 1). There were significant differences in the HRCT scores between the groups (*P* < 0.01). As shown in Fig. 2, there was a weak but significant correlation between the HRTCT score and PaO_2_/FIO_2_ ratio (ρ = − 0.258, *P* = 0.002). These findings indicated that higher HRCT score might be associated with more severe impairment of oxygenation.

The mean overall HRCT scores were 175 ± 69 in the survivors and 193 ± 71 in the non-survivors, respectively. There was no significant difference in the scores between the survivors and the non-survivors (*P* = 0.182). A receiver operating characteristic (ROC) curve determined the best cut-off value of the HRCT score of 210 for prediction of the 30-day survival. The area under the ROC curve was 0.583, and the 95% confidence interval (CI) was 0.47 to 0.70. We compared various clinical parameters and the outcome between the patients with the HRCT scores lower than 210 and those with the scores of 210 or higher (Table 3). The patients with higher HRCT scores had significantly shorter ventilator-free days than those with the scores lower than 210 (*P* = 0.020). In addition, the patients with elevated HRCT scores had significantly higher 30-day mortality (*P* = 0.04). It was indicated that the higher HRCT scores might be associated with severity of the disease and poor outcome of ARDS patients.

**Table 3:** Characteristics of ARDS patients with high and low HRCT scores

	HRCT score		
	< 210 (*n* = 104)	≥ 210 (*n* = 36)	*P* value
ARDS severity			
mild, n (%)	32 (31%)	4 (11%)	0.043*
moderate, n (%)	49 (47%)	19 (53%)	
severe, n (%)	23 (22%)	13 (36%)	
Clinical scores			
APACHE II score	23.3 ± 7.1	25.7 ± 7.0	0.083
SOFA score	11.0 ± 3.4	10.8 ± 3.9	0.83
Laboratory data			
LDH (IU/L)	355 ± 198	370 ± 191	0.69
CRP (mg/dL)	12.3 ± 9.6	11.2 ± 8.8	0.95
Lactate (mmol/L)	2.8 ± 2.3	2.4 ± 2.2	0.47
PaO_2_/FIO_2_	168.2 ± 72.6	135.9 ± 64.2	0.019
Outcomes			
30-day mortality (%)	23 (22%)	15 (42%)	0.047
Ventilator-free days	12.9 ± 10.7	8.4 ± 9.8	0.02
ICU-free days	12.0 ± 8.7	8.5 ± 8.6	0.038

*chi-square test

### Predictors of weaning failure from mechanical ventilation

Univariate logistic regression analysis was performed in the entire patients with ARDS. Age of 70 or older, HRCT score of 210 or higher, and APACHE score of 23 or greater were significantly associated with weaning failure (Table 4). Blood lactate level of 2.0 mg/dL or higher tended to decrease the likelihood of successful withdrawal from mechanical ventilation (*P* < 0.1).

**Table 4:** Predictors of weaning failure from mechanical ventilation

	Univariate		Multivariate	
	Odds ratio (95%CI)	*P* value*	Odds ratio (95%CI)	*P* value*
Female	0.66 (0.31–1.4)	0.27		
Age (≥70 y/o)	2.2 (1.1–4.2)	0.02		
Lactate ≥2.0 mmol/L	2.6 (1.3–5.3)	0.071	2.3 (1.1–5.1)	0.029
HRCT score ≥ 210	2.5 (1.1–5.3)	0.02	2.4 (1.0–5.6)	0.049
APACHE II ≥ 23	2.1 (1.1–4.2)	0.027		
Chronic heart disease	1.9 (0.8–4.3)	0.12		
Diabetes	1.3 (0.35–1.7)	0.493		

*Logistic regression analysis

From the result of multivariate logistic regression analysis, elevated level of blood lactate and increased HRCT score were independently associated with unsuccessful ventilator withdrawal [odds ratio (OR) 2.35, 95% CI 1.09–5.07, *P* = 0.029 and OR 2.38, 95% CI 1.00–5.60, *P* = 0.049, respectively].

### Predictors of 30-day mortality

Univariate logistic regression analysis indicated that male gender, older age, higher level of blood lactate, and higher HRCT score were significantly associated with 30-day mortality (Table 5). Use of catecholamines tended to decrease the likelihood of survival (*P* < 0.1).

**Table 5:** Predictors of 30-day survival

	Univariate		Multivariate	
	Odds ratio (95%CI)	*P* value*	Odds ratio (95%CI)	*P* value*
Female	0.34 (0.13–0.87)	0.024	0.25 (0.08–0.79)	0.018
Age (≥70 y/o)	3.0 (1.4–6.7)	0.005		
Lactate ≥2.0 mmol/L	3.3 (1.5–7.2)	< 0.001	3.5 (1.4–8.9)	0.092
HRCT score ≥ 210	2.4 (1.1–5.4)	< 0.001	4.3 (1.5–11.6)	0.0045
Direct lung injury	1.0 (0.42–2.60)	0.94		
Use of catecholamine	2.1 (0.8–4.3)	0.065		

*Logistic regression analysis

Based on the result of multivariate logistic regression analysis, male gender was independently associated with decreased likelihood of survival. In addition, higher level of blood lactate and higher HRCT score were also independently associated with 30-day mortality (OR 3.50, 95% CI 1.4–8.9, *P* < 0.01 and OR 4.30, 95% CI 1.5–11.6, *P* = 0.0045, respectively).

## Discussion

In the present study, we evaluated the severity criteria in the Berlin definition in consecutive patients with ARDS and showed that they might be associated with the prognosis and organ failure. Prognostic values of various clinical parameters were also assessed. Multivariate logistic regression analyses revealed that blood lactate level and HRCT score of fibroproliferative changes of the lungs were independently associated with weaning failure and 30-day mortality, suggesting that they might be predictive of the outcome of patients with ARDS.

In this study, 7.4% of the patients who were admitted to our ICU met the Berlin definition criteria of ARDS. The LUNG SAFE investigators reported that ARDS represented 10.4% (95% CI, 10.0–10.7%) of total ICU admissions [4]. They also described some geographic variation, with Asian countries having a lower incidence than Europe or North America. We considered that the incidence of ARDS in this study could be comparable with the numbers in the LUNG SAFE study.

In the Berlin definition, ARDS was stratified into three stages according to severity of oxygenation impairment at ARDS onset [1]. However, Hernu and colleagues reported that this ARDS stage was not significantly associated with patient death at day 28 [3]. They showed that the 28-day mortality was 35.0%, which amounted to 30.9% in mild, 27.9% in moderate, and 49.3% in severe categories (*P* < 0.01 between mild or moderate and severe, *P* = 0.70 between mild and moderate). In the present study, the 30-day mortality was 10.0% in mild, 23.5% in moderate, 55.0% in severe categories. Although the mortality was markedly high in severe ARDS, it was close between mild and moderate categories, which was comparable with the findings by Hernu and coworkers [3]. The severity stratification in the Berlin definition may be useful for identifying severe ARDS patients with high risk of death, but it may have less significance to differentiate between mild and moderate diseases based upon the oxygenation impairment. Thille and colleagues performed a pathological analysis comparing the presence of diffuse alveolar damage (DAD) in ARDS patients and observed that only 45% of the patients with ARDS had DAD [11]. When only patients defined as having severe ARDS were analyzed, the prevalence of DAD increased to 58%, but fell to 10 to 14% when applied to the milder cases. Concerning pathological changes, we considered that the Berlin definition might have better power in defining severe ARDS.

CT and other lung imaging techniques have been gaining a role as diagnostic tools to optimize lung assessment and ventilator management in patients with ARDS, although it remains controversial whether CT findings are predictive of outcomes [7, 12, 13]. The HRCT scores, which we estimated in this study, have been shown to represent the fibroproliferative change of the injured lung. The fibroproliferative phase of ARDS has traditionally been regarded as a late event [14]. However, Marshall and colleagues revealed increased lung collagen turnover within 24 h of diagnosis, indicating fibroproliferation is initiated early in the disease course [15]. In addition, Thille and coworkers observed proliferative phase of DAD in more than 30% of ARDS patients as early as on day 3 [16]. The development of severe fibroproliferative change has been associated with a poor prognosis with high mortality and/or prolonged ventilator dependency [17]. Ichikado and colleagues reported that higher HRCT score at diagnosis of ARDS was an independent predictor for death and ventilator dependency regardless of the cause of ARDS [10]. They also showed that oxygenation was poorer in the group with higher HRCT score. In this study, the HRCT scores were not significantly different between the surviving and non-surviving patients, but the score of 210 or higher was independently associated with ventilator withdrawal failure and 30-day mortality, which was compatible with the previous report. In addition, the cut-off value of the HRCT scores we determined was identical with the number described by Ichikado and colleagues [9]. It was suggested that the scoring system of HRCT images could be generalized.

In the present study, multivariate analyses revealed that both the HRCT score and blood lactate levels were associated with the outcome of the ARDS patients although there could be no direct relationship between the two indicators. The HRCT score represents fibroproliferative change in the lung pathology, whereas blood lactate levels are associated with tissue hypoxia [18]. An elevated level of lactate has been shown to be associated with multiple organ failure (MOF) or septic shock [19, 20]. Ferring and Vincent showed that MOF was the principal cause of death, rather than respiratory failure [21]. Considering that MOF is the major cause of death of ARDS patients, the prognostic value of blood lactate seems reasonable. Whereas HRCT is sometimes difficult to conduct especially in severely ill patients, blood lactate level, which can be measured along with arterial blood gases, could be a convenient indicator.

The present study has some potential limitations. First, this study is based on the data from a single ICU in a university hospital. Second, in this study, sepsis was defined with the Surviving Sepsis Campaign guideline in 2012 [22]. In the current definition of sepsis, organ dysfunction is more focused [23]. As blood lactate levels are associated with tissue hypoxia, further studies should be conducted to examine the significance of lactate levels using the current definition of sepsis. Third, there might be some patients with pre-existing chronic interstitial lung diseases. We carefully excluded such patients from the HRCT analysis by history taking, imaging data available before onset of ARDS and the presence of coarse reticulation and honeycombing on HRCT images. However, pre-existing interstitial changes cannot be completely denied in some patients, especially in those without previous imaging data. Fourth, because we collected the data from the medical records written by a number of medical staffs, the direct causes of death could not be specified. Fifth, the statistical significances were weak about both HRCT score and blood lactate, which could be subject to further investigation.

## Conclusions

In the present study, we found that the severity criteria in the Berlin definition might be associated with the prognosis and organ failure in Japanese patients with ARDS. It was also revealed that blood lactate level and HRCT score of fibroproliferative changes of the lungs were independently associated with ventilator dependency and 30-day mortality, suggesting that they might be predictive of the outcome of ARDS patients including those without sepsis.

## List of abbreviations

APACHE: Acute physiology and chronic health evaluation
ARDS: Acute respiratory distress syndrome
BMI: Body mass index
CI: confidence interval
CRP: C-reactive protein
HRCT: High-resolution computed tomography
ICU: Intensive care unit
LDH: Lactate dehydrogenase
MOF: Multiple organ failure
OR: Odds ratio
ROC: Receiver operating characteristic
SAPS: Simplified acute physiology score
SD: Standard deviation
SOFA: Sequential organ failure assessment
